# Vertical Transmission of Respiratory Syncytial Virus Modulates Pre- and Postnatal Innervation and Reactivity of Rat Airways

**DOI:** 10.1371/journal.pone.0061309

**Published:** 2013-04-18

**Authors:** Giovanni Piedimonte, Cheryl Walton, Lennie Samsell

**Affiliations:** 1 Pediatric Institute and Children’s Hospital, The Cleveland Clinic, Cleveland, Ohio, United States of America; 2 Department of Pediatrics, West Virginia University, Morgantown, West Virginia, United States of America; University of Georgia, United States of America

## Abstract

**Background:**

Environmental exposure to respiratory syncytial virus (RSV) is a leading cause of respiratory infections in infants, but it remains unknown whether this infection is transmitted transplacentally from the lungs of infected mothers to the offspring. We sought to test the hypothesis that RSV travels from the respiratory tract during pregnancy, crosses the placenta to the fetus, persists in the lung tissues of the offspring, and modulates pre- and postnatal expression of growth factors, thereby predisposing to airway hyperreactivity.

**Methodology:**

Pregnant rats were inoculated intratracheally at midterm using recombinant RSV expressing red fluorescent protein (RFP). Viral RNA was amplified by RT-PCR and confirmed by sequencing. RFP expression was analyzed by flow cytometry and viral culture. Developmental and pathophysiologic implications of prenatal infection were determined by analyzing the expression of genes encoding critical growth factors, particularly neurotrophic factors and receptors. We also measured the expression of key neurotransmitters and postnatal bronchial reactivity in vertically infected lungs, and assessed their dependence on neurotrophic signaling using selective biological or chemical inhibition.

**Principal Findings:**

RSV genome was found in 30% of fetuses, as well as in the lungs of 40% of newborns and 25% of adults. RFP expression was also shown by flow cytometry and replicating virus was cultured from exposed fetuses. Nerve growth factor and its TrkA receptor were upregulated in RSV- infected fetal lungs and co-localized with increased cholinergic innervation. Acetylcholine expression and smooth muscle response to cholinergic stimulation increased in lungs exposed to RSV *in utero* and reinfected after birth, and blocking TrkA signaling inhibited both effects.

**Conclusions/Significance:**

Our data show transplacental transmission of RSV from mother to offspring and persistence of vertically transmitted virus in lungs after birth. Exposure to RSV *in utero* is followed by dysregulation of neurotrophic pathways predisposing to postnatal airway hyperreactivity upon reinfection with the virus.

## Introduction

Respiratory syncytial virus (RSV) is the most common cause of lower respiratory tract infections in infants and young children, and strong epidemiologic evidence suggests that early- life infections with this virus predispose to chronic respiratory dysfunction and asthma, possibly related to persistence of the virus itself or to its effects on lung development [1]. Although this virus targets primarily the bronchiolar epithelium [2], several observations indicate that RSV can spread to extra-pulmonary sites and have systemic implications both in animal models [3], [4] and in humans [5], [6]. Also, our more recent studies suggest that RSV acquired during infancy can persist latently in cells providing an immunologically privileged sanctuary [7].

In its intra- and extra-pulmonary targets, RSV has been shown to modulate the biological effects of neurotrophins, a family of proteins that play a key role in neuronal survival, development, and function [8]. In particular, the prototypical nerve growth factor (NGF) [9], [10] controls the expression of key neurotransmitters and their release from peripheral neurons [11]. In addition, NGF has both direct and indirect (i.e., nerve-mediated) effects on innate and adaptive immunity and has been associated with allergic inflammation in animal models as well as in humans [12], [13]. Finally, NGF prevents cell death by increasing expression of the anti- apoptotic *Bcl*-2 family members, thereby functioning as an autocrine/paracrine factor that keeps infected bronchial epithelial cells alive to support viral replication [2].

It is generally accepted that the pathophysiology of RSV bronchiolitis is driven by the inflammatory response mounted by the child’s immune system against virus acquired by *horizontal* (i.e., interpersonal) transmission in the first months after birth. Whether RSV can cross the placental barrier and interact directly with the developing lungs of the fetus has never been entertained and, to the best of our knowledge, there are no reports of *vertical* transmission of RSV in animal models or in humans. Yet, a number of infectious agents, including flaviviruses, herpesviruses, retroviruses [14], and even orthomyxoviruses like the H5N1 avian influenza virus [15] have been shown to cross the placenta and establish persistent infection of the offspring. Furthermore, viral infections during pregnancy have been linked to chronic diseases generally considered of non-infectious etiology (e.g., autism) [16], but have not been adequately explored for asthma and other chronic lung diseases.

Therefore, we investigated the presence of vertically transmitted RSV in fetal tissues and in the lungs of offspring delivered from rat dams infected at midterm. Developmental and pathophysiologic implications of prenatal infection were studied analyzing the expression of genes encoding critical growth factors, particularly neurotrophic factors and their cognate receptors. Lastly, we measured the expression of key neurotransmitters and postnatal bronchial reactivity in vertically infected lungs, and assessed their dependence on neurotrophic signaling using selective biological or chemical inhibition.

## Methods

### Ethics Statement

All experimental procedures followed in this study were conducted according to relevant national and international guidelines and were approved by the West Virginia University Institutional Animal Care and Use Committee.

### Animals

Non-pregnant adult (10 weeks of age) pathogen-free Fischer 344 (F-344) rats were purchased from Harlan Sprague Dawley (Indianapolis, IN) and housed in our transgenic barrier facility using polycarbonate isolation cages mounted on racks that provided positive individual ventilation with class-100 air at the rate of 1 cage change of air per minute (Techniplast, Buguggiate, Italy). Bedding, water, and food were autoclaved before use and all manipulations were conducted inside class-100 laminar flow hoods. These rats were mated overnight using 2 females per male, and the day on which spermatozoa were found in the vaginal smear was recorded as day 1 of gestation [17]. On day 12 of gestation, approximately corresponding to midterm, the dams were transferred to a separate room for inoculation with RSV suspension or with virus-free medium. Afterwards, we used separate rooms for infected and pathogen-free rats, both of which were serviced by specifically trained husbandry technicians.

### RSV Preparation

Rats used in this study were infected with a recombinant RSV-A_2_ strain expressing the RFP gene (rrRSV), kindly provided by Dr. Mark Peeples (Columbus Children Research Institute, Columbus, OH) and Dr. Peter Collins (National Institutes of Health, Bethesda, MD) [18]. This virus was generated from the full-length rgRSV plasmid MP224 by replacing its first gene, which encodes the enhanced green fluorescent protein, with the wild-type *Discosoma* RFP gene from pDsRed (Clontech, Palo Alto, CA). To accomplish this, the *Bst*XI site within the RFP gene was disrupted by PCR mutagenesis and the modified RFP gene was amplified by PCR with primers that added *Bst*XI sites to each end, in addition to the gene start and NS1 untranslated region preceding the RFP gene and the L gene end following the RFP gene. The PCR product was inserted into the *Bst*XI site of D13, a partial RSV clone containing the 3′ RSV genome end. The *Age*I/*Spe*I fragment of this clone (MP230) was transferred into the full-length clone (MP224), which had been digested completely with *Age*I and partially with *Spe*I. The resulting construct (BN1) was rescued as replicating virus by co-transfection of BN1 and 4 plasmids producing the proteins needed to initiate RSV replication and transcription into HeLa cells infected with the recombinant vaccinia virus MVA-T7.

The rrRSV stock was propagated using HEp-2 cells grown at 37°C and 5% CO_2_ in Eagle’s minimum essential medium (MEM) supplemented with 10% fetal bovine serum (FBS; American Type Culture Collection, Manassas, VA) and 1% each of Glutamax, penicillin/streptomycin, and 4-(2-hydroxyethyl)-1-piperazineethanesulfonic acid (HEPES; Invitrogen, Grand Island, NY). Cells at approximately 50% confluence were inoculated with 3 ml per dish of virus stock diluted 1/10 with heat-inactivated FBS. After incubation for 2 h at 37°C, the inoculum was removed and replaced with 25 ml of fresh medium. Medium was replaced again 2 days later, and the virus was harvested after 1–2 additional days of incubation, at which point all cells appeared bright red when viewed with a fluorescent microscope.

To harvest the virus, infected cells were scraped from the plate, separated with a pipette, mixed at medium speed, and pelleted by centrifugation at 1,200 *g* for 5 min. The supernatant was collected and cell debris removed by centrifugation at 9,500 *g* for 20 min in a centrifuge refrigerated at 4°C. One-ml aliquots of the supernatant were snap-frozen in liquid nitrogen and stored at −70°C until use. The final titer was determined with a modified plaque-forming unit assay [2]. Supernatants and cell lysates from virus-free flasks of HEp-2 cells in MEM were harvested, centrifuged, and frozen in aliquots following the same protocol to obtain the virus-free medium used as sham infection control.

### RSV Infection

Pregnant dams were lightly anesthetized with methohexital sodium (25 mg/kg, i.p.) and inoculated with 1.9×10^6^ TCID_50_ of rrRSV suspension by intratracheal instillation to establish a lower respiratory tract infection. Control pathogen-free dams were dosed with an equal volume of virus-free medium using the same procedure. Whole fetuses were harvested 5 days after the inoculation of their mother, i.e., on embryonic day 17 (E17). RSV-infected and pathogen-free dams were deeply anesthetized with sodium pentobarbital (50 mg/kg, i.p.) and both horns and the cervix of the gravid uterus were tied off with silk suture thread. The uterus was carefully removed and rinsed twice in a sterile tissue culture plate containing 50 ml of saline. The uterus was then opened with sterile scissors and forceps and a separate set of scissors and forceps were used to remove each fetus from its placenta and place it in a sterile cryovial, which was immediately frozen in liquid nitrogen and stored at −70°C.

Lung tissues from the offspring of both RSV-infected and pathogen-free dams were collected at 24 h after birth (newborns), 2 weeks of age (weanlings), or 10 weeks of age (adults). The pups were delivered at full term and immediately transferred to pathogen-free surrogate mothers. After euthanasia with sodium pentobarbital (75 mg/kg, i.p.) followed by exsanguination, the chest was opened and the lungs were removed, placed in a sterile cryovial, frozen in liquid nitrogen, and stored at −70°C.

### RT-PCR

The frozen specimens were homogenized in RLT lysis buffer using a Mini Beadbeater homogenizer (Biospec Products, Bartlesville, OK). Total RNA was extracted from the homogenates using RNeasy Mini-Kits (Qiagen, Hilden, Germany) according to the manufacturer’s specifications. PCR was performed using the OneStep RT-PCR kit (Qiagen). Briefly, RNA samples (1–10 ng) were added to a 50 µl master mix consisting of 400 µM each of deoxynucleotide triphosphates, 10 units of RNAse inhibitor, 2 µl of OneStep RT-PCR enzyme mix, and 50 pmol each of primers flanking the nucleotide sequence for RSV; NGF and brain- derived neurotrophic factor (BDNF); tropomyosin-related kinase A (TrkA) and B (TrkB) high-affinity receptors for NGF and BDNF respectively; p75^NTR^ pan-neurotrophin receptor; epidermal growth factor and its receptor (EGF/EGFR), transforming growth factor beta (TGF-β), vascular endothelial growth factor (VEGF); and the housekeeping gene β-actin. The same master mix without the RNA sample was used as a negative control.

The primers pairs shown in **Table 1** were designed on the basis of previously published protocols to discriminate cDNA-generated PCR products from genomic DNA contamination [19]. RSV primers (5′-GCGATGTCTAGGTTAGGA-3′; and 5′-GCTATGTCCTTGGGTAGT-3) target the sequence 1303–1712 bp of the RSV serotype A_2_ genome (GenBank accession number M11486), which encodes for the viral nucleocapsid (N) protein. Because the GenBank sequence is missing 34 nucleotides in the 3′ leader region, the position of the target sequence on the complete RSV-A_2_ genome is 1347–1756. The N protein is a structural protein located inside the virion tightly bound to the RNA strand, and is typically targeted to detect the presence of whole virus in infected cells. No-template negative controls were run every time a PCR reaction was set up, and RSV-infected cells were used as a positive control.

**Table 1:** RT-PCR primers and relative targets.

*Target*	*Sense aa*	*Anti-sense*	*Size bp*	*Accession number*	*Gene ID*	*Sense nucleotides*	*Anti-sense nucleotides*
RSV	71–75	201–205	409	DQ7805631	11259	5′-GCG ATG TCT AGG TTA GGA-3′	5′-GCT ATG TCC TTG GGT AGT-3′
NGF	100–105	225–230	395	M36589	310738	5′-CTG GAC TAA ACT TCA GCA TTC-3′	5′-TGT TGT TAA TGT TCA CCT CGC-3′
BDNF	129–134	230–236	293	M61178	24225	5′-AGC TGA GCG TGT GTG ACA GTA T-3′	5′-GTC TAT CCT TAT GAA CCG CCA G-3′
TrkA	138–145	354–360	690	M021589	59109	5′-GCC TTC GCC TCA ACC AGC CCA-3′	5′-CTC TTG ATG TGC TGT TAG TGT-3′
TrkB	42–49	251–260	664	M55291	25054	5′-OH CCG CTA GGA TTT GGT GTA CTG AGC CTT CT-3′	5′-OH CCA CTG TCA TCA GAT GAA ATG TTC GTT ATC CT-3′
p75^NTR^	60–65	274–279	663	M012601	24596	5′-OH AGC CAA CCA GAC CGT GTG TG-3′	5′-OH TTG CAG CTG TTC CAC CTC TT-3′
β-actin	216–223	369–375	285	C005111.2	81822	5′-OH TCA TGA AGT GTG ACG TTG ACA TCC GT-3′	5′-OH CTT AGA AGC ATT TGC GGT GCA CGA TG-3′

Amplification was performed using a GeneAmp PCR System 9600 thermal cycler (Perkin- Elmer, Waltham, MA). The process was started with an initial step of 50°C/30 min, then 95°C/15 min, followed by 30–45 cycles with a denaturing step followed by an annealing step, an extension step, and one final extension step at 68–72°C for 10 min. All programs included a 4°C hold step at the end. Amplified PCR products were size-fractionated by electrophoresis through a 2% agarose gel, stained with ethidium bromide, and photographed using an imaging system (FOTO/Analyst Luminary Workstation, Fotodyne, Hartland, WI). The intensity of DNA bands was analyzed by computerized densitometry (TotalLab TL-101 Image Analysis Software, Nonlinear Dynamics, Durham, NC), and expressed as the ratio of the densitometry score measured for each target normalized by the β-actin control.

### RSV Sequencing

In every sample with PCR-detectable viral RNA, amplicons identity was confirmed by sequence analysis. Bands were excised from the agarose gel and purified using the MinElute Gel Extraction kit (Qiagen). The purified product was quantified (Nanodrop, Wilmington, DE) and sequenced at MWG Biotech (High Point, NC). We then analyzed these sequences for homology with the human RSV-A_2_ genome using the Geneious software (version 3.4.5; Biomatters, Auckland, New Zealand) and the *blastn* algorithm, which compares alignment and homology of the query sequence against nucleotide sequences from the database of the National Center for Biotechnology Information. A bit score derived from the raw alignment score (sum of substitution and gap scores) was used to calculate the expectation value (E-value) expressing the probability of a false positive for each sequence.

### Histopathology

Rat fetuses were fixed in 10% neutral-buffered formalin, bisected on the sagittal midline, processed overnight, and sagittally paraffin embedded. Newborn lungs were similarly fixed in 10% formalin and coronally paraffin embedded. Five-µm hematoxylin and eosin stained sections from each fetus and newborn lung were evaluated by two expert pathologists blinded to the treatment protocol using routine light microscopy.

### FACS

Fetuses harvested for cytometry detection of RFP and NGF were placed into ice-cold Dulbecco’s MEM (DMEM) with 10% FBS and crushed between two slides to obtain a single- cell suspension, which was then filtered through a 70-µm cell strainer to eliminate larger cellular debris. Red blood cells were lysed in 3 ml of 1X PharmLyse buffer (BD Biosciences, San Jose, CA) for 3 min at room temperature, and the residual cells were pelleted in ice-cold DMEM, washed twice with phosphate-buffered saline with 2% FBS and 0.02% sodium azide (PBS-Az), and resuspended in 10% formaldehyde for 30 min. Cells were washed again with PBS-Az and incubated on ice in 70% ice-cold ethanol followed by an additional wash in PBS-Az. Non- specific binding was blocked with an additional incubation on ice with whole rat IgG (Jackson ImmunoResearch, West Grove, PA) in PBS-Az for 15 min. A polyclonal rabbit anti-NGF antibody or the corresponding control isotype was added and incubated for an additional hour. Lastly, the cells were washed twice and incubated with Alexa Fluor 488 donkey anti-rabbit antibody for 30 min. Data were acquired using a FACS Calibur instrument and analyzed with the CellQuest Pro software (BD Biosciences).

### Viral Culture

To determine if vertically transmitted RSV not only replicates, but also generates mature virions able to spread the infection to neighboring cells, we incubated HEp-2 cells with extracts prepared by filtration with 0.2-µm membranes of fetal tissues from rrRSV-infected or control dams. After 24 h, the extracts were removed and the cells washed twice with medium. Cells were observed with a fluorescence microscope at 24 h and 48 h of incubation to detect infection by vertically transmitted RFP-tagged RSV.

### Immunochemistry

To visualize NGF expression in HEp2 cells exposed to fetal extracts, the cells were rinsed in PBS and fixed in 4% methanol-free formaldehyde at room temperature for 10 min, and then permeabilized with 0.5% Triton X-100 for 20 min. After incubation for 20 min in PBS/5% BSA to block non-specific antibody binding, the cells were stained on coverslips for 1 h with a polyclonal rabbit anti-NGF antibody diluted in PBS/5% BSA (Santa Cruz Biotechnology, Santa Cruz, CA) or with the corresponding control isotype, followed by Alexa Fluor 488 donkey anti-rabbit secondary antibody (Invitrogen) for 30 min. The coverslips were then inverted on slides and mounted using Prolong Gold anti-fade reagent with 4′,6-diamidino-2- phenylindole (DAPI; Invitrogen). Images were obtained using a Zeiss LSM510 confocal microscope with an AxioImager Z1 system (Carl Zeiss, Jena, Germany). In the same preparations, the PE channel allowed to localize the RFP expressed by rrRSV-infected cells.

To localize the relative distribution of rrRSV infection and NGF expression in fetal tissues, sections fixed and permeabilized as described above were stained on coverslips for 1 h with a polyclonal goat anti-NGF antibody (Santa Cruz Biotechnology) and a polyclonal rabbit anti-RFP antibody (Abcam, Cambridge, MA), or with the corresponding control isotypes, followed by Alexa Fluor 488 donkey anti-rabbit antibody and Alexa Fluor 647 donkey anti-goat antibody (Invitrogen) for 30 min. The coverslips were mounted on slides and imaged as described above.

To localize adrenergic, cholinergic, and non-adrenergic non-cholinergic (NANC) nerves in fetal tissues, frozen sections were fixed in cold acetone for 10 min at 4°C, washed 3 times with PBS, and stained for 1 h with one of the following primary antibodies: a. polyclonal rabbit anti- noradrenaline (Abcam); b. polyclonal rabbit anti-acetylcholine (AbD Serotec, Raleigh, NC); or c. polyclonal goat anti-substance P (Santa Cruz Biotechnology). These slides were also co-stained for 1 h at room temperature with a polyclonal goat or rabbit anti-NGF antibody (Santa Cruz Biotechnology), followed by Alexa Fluor 488 donkey anti-rabbit antibody and Alexa Fluor 647 donkey anti-goat antibody (Invitrogen) for 45 min in 1.5% FBS.

### Neurotransmitters

Lung tissues were homogenized in 5 volumes of lysis buffer containing Tris–HCl (20 mM), NaCl (137 mM), NP-40 (1%), glycerol (10%), phenylmethylsulfonyl fluoride (1 mM), aprotinin (10 mg/ml), leupeptin (1 mg/ml), and sodium vanadate (0.5 mM) and were centrifuged at 14,000 rpm for 10 min. Acetylcholine, noradrenaline, and substance P levels in the supernatants were measured by ELISA using commercially available kits (MyBioSource, San Diego, CA). In short, samples and standards were transferred to antibody-coated 96-well plates and incubated with a horseradish peroxidase-tagged second antibody for 1 h at 37°C. Plates were then washed 5 times with buffer, chromogenic substrates were added to each well, and the reaction was stopped after 15 min with an acidic solution. Absorbance was measured at 450 nm wavelength and the final concentration was extrapolated from a standard curve.

### Airway Reactivity

Ten-day old rats delivered from pathogen-free or RSV-infected dams were given either sterile medium (C+C and R+C groups) or RSV suspension (C+R and R+R groups) by intratracheal instillation. All four groups of pups were delivered, housed, fed, and anesthetized exactly in the same way. Airway smooth muscle responsiveness was evaluated 5 days later by measuring: a. contractile force generated by tracheal strips exposed to transmural electrical field stimulation (EFS) *in vitro*; or b. change in respiratory system resistance (R_rs_) and compliance (C_rs_) in response to increasing concentrations of inhaled methacholine *in vivo*. The former methodology evaluates smooth muscle contraction resulting from the release of endogenous neurotransmitters by intrinsic postganglionic vagus nerve terminals innervating the airways, whereas the latter primarily measures airway smooth muscle responses to exogenously delivered cholinergic agonist.

For *in vitro* experiments, the tracheas were bathed in ice-cold modified Krebs-Henseleit solution containing 95% O_2_ and 5% CO_2_. Two cartilage ring-wide segments were cut, opened opposite of the smooth muscle, and tied to secure one end of the tissue to an isometric force transducer and the other end to the bottom of the bath. Organ baths were maintained at 37°C by an external water bath and tissues were placed under 0.04 g of resting tension. The tracheal strips were mounted between a set of platinum electrodes to deliver EFS. Frequency response curves were generated by increasing the frequency from 0.1 to 30 Hz, using a sub-maximal voltage of 120 V, 10-ms pulse duration, and 10-s square wave trains. Responses were expressed as change in contractile force (g) from baseline.

For *in vivo* experiments, pups were anesthetized with methohexital sodium (50 mg/kg, i.p.) and their trachea was intubated with an 18-gauge catheter inserted in a small cut below the cricoid cartilage. The catheter was then connected to a nebulizer attached to a Flexivent® system (Scireq, Montreal, Canada) to obtain accurate measurements of respiratory mechanics and airway function. R_rs_ and C_rs_ were first measured at baseline (i.e., after nebulization of sterile PBS) and then after nebulization of increasing concentrations of methacholine ranging from 0.5 to 8.0 mg/ml.

### NGF Inhibition

To test the effects of selective neurotrophin inhibitors on the synthesis of key neurotransmitters and on the airway reactivity to methacholine *in vivo*, we used two different strategies: a. immunologic inhibition with a specific NGF-blocking antibody; or b) chemical inhibition of receptor tyrosine kinase with the specific antagonist K252a. The antibody or antagonist was injected subcutaneously 60 min before RSV inoculation, then daily for 4 days, and finally 60 min before the methacholine challenge on day 5.

As neurotrophic factors are highly conserved in different species [20], we blocked endogenous NGF activity using a polyclonal rabbit anti-mouse antibody (16 mg/kg i.p.; Abcam), and used rabbit purified IgG as an isotype control. Timing and doses of the anti-NGF treatments were chosen on the basis of previous studies that confirmed the efficacy of this antibody in blocking NGF biological activity in rats [21].

For receptor tyrosine kinase inhibition, rats were injected with K252a (50 µg/kg i.p.; Biomol, Plymouth Meeting, PA), a carbazole alkaloid that inhibits the phosphorylation of both TrkA and TrkB receptors [22] and has been shown to prevent NGF-dependent substance P production *in vitro* [23] and *in vivo* [24]. Controls were given an equal volume of vehicle (2% dimethyl sulfoxide in 0.9% NaCl). Biologically active, non-toxic doses of this inhibitor were chosen based on previously published work in our rat model [25].

### Statistical Analysis

All data are expressed as mean ± SEM. Densitometry scores from RT-PCR experiments are the average of 3 or 4 independent assays performed in duplicate. Geometric mean fluorescent intensity (MFI) was averaged from the flow cytometry measurements obtained in 3 or 4 independent experiments. RT-PCR and ELISA data were analyzed by 2-way analysis of variance (ANOVA), and if a significant effect was present post-hoc multiple pairwise comparisons with the Holm-Sidak method were used to identify significant differences between and within groups. This statistical analysis was performed using the software SigmaStat version 3.5 (Systat, Point Richmond, CA).

For the analysis of airway reactivity data, a linear mixed model approach using restricted maximum likelihood was employed to account for the repeated measures in each rat [26]. This approach incorporates incomplete cases permitting the best use of all available data [26], [27]. The R_rs_ outcome was regressed against dam-pups group (C+C, R+C, C+R, and R+R), methacholine dose (mg/ml), a squared term for dose, and an interaction between dose and dam-pups group. The within-rat correlation was modeled using a continuous autoregressive correlation structure of order 1 accounting for each rat’s correlated outcome measures, and the variation was modeled with homoscedastic within-rat errors [26], [28].

The C_rs_ outcome was regressed against dam-pups group, methacholine dose, the natural logarithm of dose, an interaction between dose and dam-pups group, and an interaction between the natural logarithm of dose and dam-pups group. The within-rat correlation was modeled using a continuous autoregressive correlation structure of order 1 accounting for each rat’s correlated outcome measures, and the variation was modeled with different variances for each dose level [26], [28]. A random effect for dose was included in the model. Model-based mean trends and 95% confidence intervals for each of the dam-pups groups were estimated. Model selection considered the agreement across both the Akaike and Bayesian information criterion, favoring the retention of all aforementioned variables. Residual analysis showed satisfactory support for the normality assumption. The statistical software “R” was used for this analysis [28].

## Results

### Vertical Transmission of RSV

**Figure 1:**
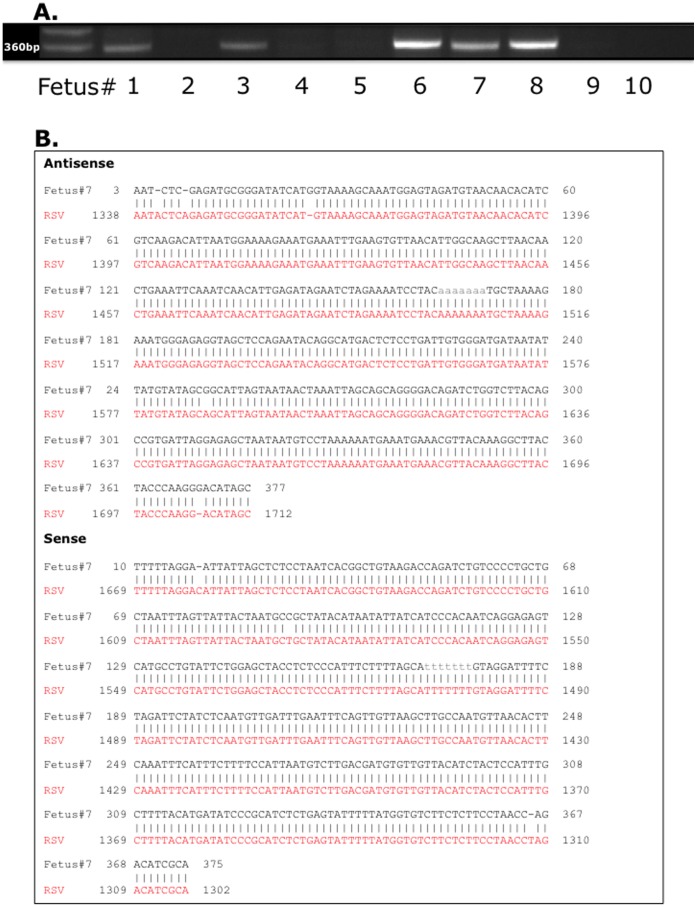
Vertical transmission of RSV genome. *(* ***A*** *)* RT-PCR analysis of fetuses harvested from RSV-infected dams using primers specific for the viral nucleocapsid protein. The bands in the first lane of the gel are DNA molecular weight standards; each of the other lanes shows RNA amplified from an individual fetus identified by the number indicated below the gel. RSV genomic sequences were found in one-third of the fetuses (#6, 7, and 8), whereas all matched control fetuses from pathogen-free dams were negative (not shown). *(* ***B*** *)* Sequence analysis of the amplicon from Fetus #7. All positive specimens yielded a 367-bp product whose sequence shared 98% homology with the RSV-A_2_ genome.

We first sought to determine whether maternal RSV infection contracted during gestation is vertically transmitted to the fetus and whether viral sequences are harbored in the lungs of the offspring after birth. RT-PCR analysis with primers specific for the N protein of human RSV-A_2_ revealed the presence of RSV genomic sequences in 3 out of 10 (30%) fetuses harvested at day E17 from RSV-infected dams (**Figure 1A**), whereas all matched control fetuses (n = 9) from pathogen-free dams were negative. In newborns delivered by RSV-infected dams, viral RNA was detected in the lungs of 4 out of 10 animals (40%), whereas all age-matched pathogen-free controls (n = 8) were negative. In weanling rats born to RSV-infected dams and kept in a barrier facility until 2 weeks of age, viral RNA was detected in 2 out of 12 animals (17%), and all age-matched pathogen-free controls (n = 10) were negative. In adult rats born to RSV-infected dams and kept in a barrier facility until 10 weeks of age, viral RNA was detected in 2 out of 8 animals (25%), and again all age-matched pathogen-free controls (n = 8) were negative. As summarized in **Table 2**, all positive specimens yielded a 367±4 bp product whose sequence shared 98±0.4% homology with the RSV-A_2_ genome, with expected probability of a false positive based on the bit score equal or close to zero for all samples. Sequence analysis of the amplicon from Fetus #7 is shown as an example (**Figure 1B** **)**.

**Table 2:** Sequence homology between the RSV-A_2_ N-protein gene and the PCR products amplified from fetal and offspring tissues exposed to the virus *in utero*.

Sample	%Identity	Bit score	E-value	Length
***Fetus #6***	98.1	656.68	0E+00	378
***Fetus #7***	98.7	665.91	0E+00	377
***Fetus #8***	98.1	656.68	0E+00	378
***Newborn lung #2***	95.4	606.82	2E−170	367
***Newborn lung #4***	98.4	660.37	0E+00	377
***Newborn lung #5***	97.8	636.37	2E−169	370
***Newborn lung #10***	99.2	676.99	0E+00	377
***Weanling lung #3***	96.4	573.39	2E−160	360
***Weanling lung #7***	98.9	601.15	8E−169	348
***Adult lung #2***	98.6	614.43	1E−172	367
***Adult lung #5***	99.7	574.76	8E−161	335

***Legend:***

***% Identity:*** Extent to which two nucleotide sequences are invariant.

***Bit score:*** Value derived from the raw alignment score (sum of substitution and gap scores), in which the statistical properties of the scoring system used have been taken into account allowing for comparisons across sequence analysis programs.

***E-value*** *(* ***expectation value*** *)* ***:*** Probability of a false positive based on the bit score.

***Length:*** Length of target sequence homologous with unknown sequence.

**Figure 2:**
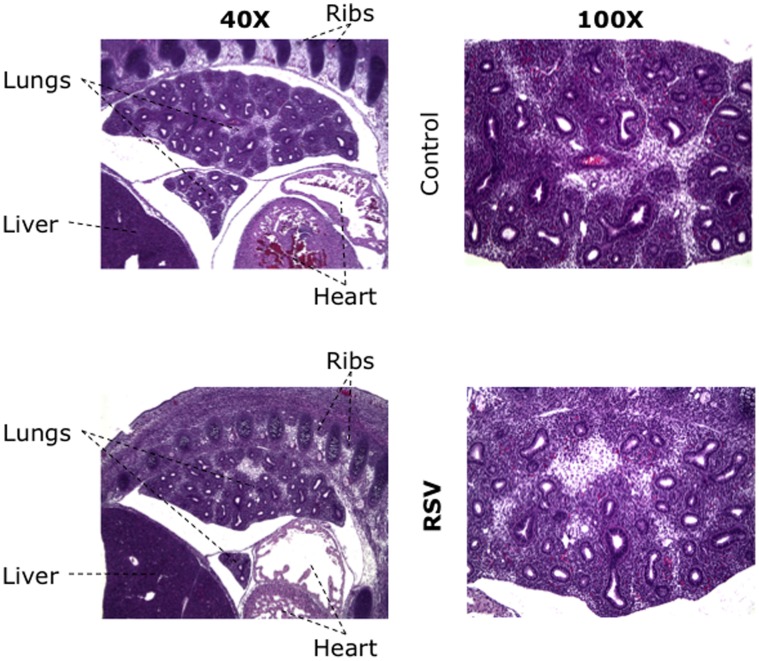
Histopathology of RSV-infected fetal lungs. Sagittal sections of an RSV- infected E17 rat fetus stained with hematoxylin and eosin, showing the lungs at two different magnifications (**40X** and **100X**). Histological analysis revealed late pseudoglandular development of the bronchial tree, which was appropriate for gestational age, and absence of acute or chronic inflammatory cell infiltrates. Similarly, the aerated newborn lung bronchi and alveolar air spaces were essentially normal without acute or chronic inflammatory cell infiltrates, and no definitive histologic differences were identified between the pups delivered from RSV-infected dams and those from pathogen-free dams (not shown).

Intrauterine exposure to RSV did not cause significant changes in fetal morphology, developmental pattern, and birth weight both in males (5.2±0.2 vs. 5.4±0.3 g; p = 0.7) and in females (4.9±0.1 vs. 5.4±0.3 g; p = 0.1). Also, it did not affect fetal viability and parity, as the average litter size observed in RSV-infected dams was similar to pathogen-free controls (9±1 vs. 8±1). For both the RSV-exposed and control groups, the fetal lungs histologically showed late pseudoglandular development of the bronchial tree, which was appropriate for gestational age (**Figure 2**). The intervening mesenchyme was essentially unremarkable, without acute or chronic inflammatory cell infiltrates. Similarly, the aerated newborn lung bronchi and alveolar air spaces were essentially normal without acute or chronic inflammatory cell infiltrates, and no definitive histologic differences were identified between the pups delivered from RSV-infected dams and those from pathogen-free dams.

Importantly, a significant antibody response was measured in the serum of all dams inoculated with RSV (anti-RSV IgG titer = 14.7±8.8 µg/ml), confirming the establishment of robust maternal infection in our Fischer rat model. None of the pathogen-free control dams had measurable serum antibodies against RSV. Also, pups exposed to RSV *in utero* did not have measurable RSV antibodies in serum samples collected after birth.

**Figure 3:**
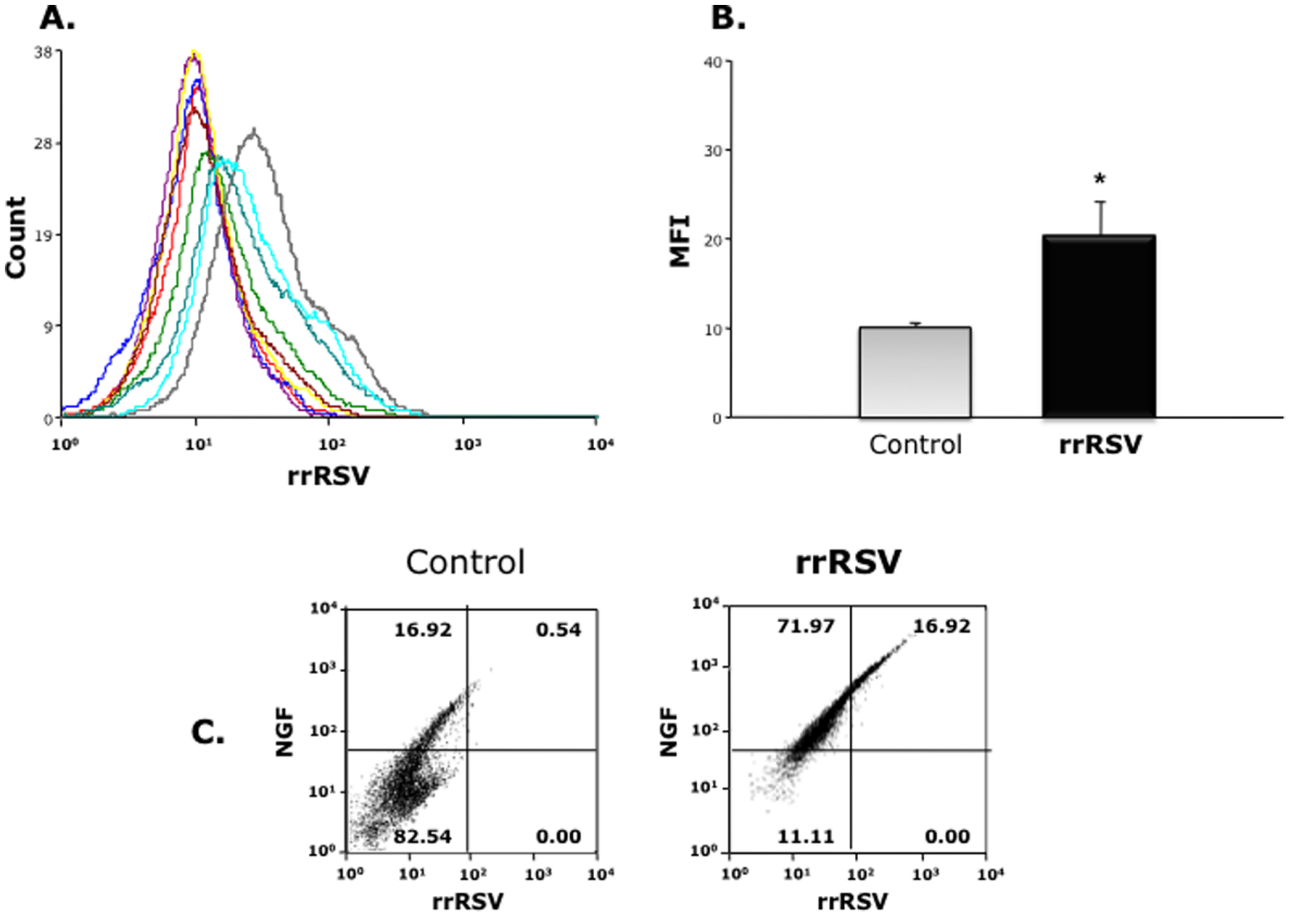
Replication of vertically transmitted RSV. *(* ***A*** *)* Fluorescence-activated sorting of fetal cells from dams infected with rrRSV or pathogen-free control dams. Each curve has a different computer-generated color corresponding to a different fetus. RFP was detected in two-thirds of the fetuses harvested from dams infected with rrRSV, whereas the other third overlapped the control fetuses harvested from pathogen-free dams. *(* ***B*** *)* Geometric mean fluorescent intensity (MFI) averaged from the same flow cytometry measurements confirmed a significant increase in red fluorescence in fetuses from rrRSV- infected dams. * = p<0.05 compared to control fetuses from pathogen-free dams. *(* ***C*** *)* FACS analysis showed that exposure to rrRSV *in utero* was associated to fetal overexpression of NGF protein.

To evaluate the infectivity of vertically transmitted RSV, we screened fetal cells exposed to rrRSV *in utero* using FACS to detect and quantify RFP gene expression, which requires active viral replication. Four out of 6 fetuses (67%) harvested from dams infected with rrRSV expressed significantly increased RFP fluorescence (**Figure 3A**), whereas the other 2 overlapped the control fetuses harvested from pathogen-free dams (n = 4). Geometric mean fluorescent intensity (MFI) averaged from the same flow cytometry measurements confirmed a significant increase of red fluorescence in fetuses from rrRSV-infected dams (p<0.05; **Figure 3B**). Whereas only 17.5% of cells from the pathogen-free fetuses expressed detectable levels of NGF protein, this population increased to 88.2% in the fetuses harvested from rrRSV-infected dams, and the expression of NGF protein was directly proportional to the titer of RFP-expressing virus (**Figure** **3C** **)**.

**Figure 4:**
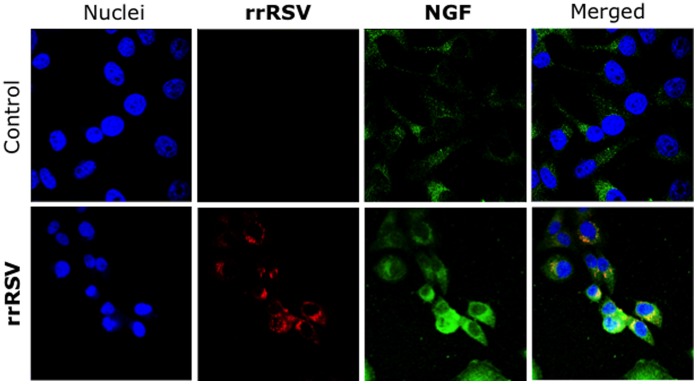
Propagation of vertically transmitted RSV. Extracts of whole fetuses delivered from dams inoculated with rrRSV or from pathogen-free controls and co-cultured with human airway epithelial cells. After 48 h of incubation, the cells were then stained with a polyclonal anti-NGF antibody. The micrographs show red fluorescence in the cytoplasm of cells exposed to fetal extracts from rrRSV-infected dams, confirming the presence of actively replicating infectious virus that is associated with markedly increased green NGF immunoreactivity. As RFP expression reflects virus infection and production of the transgene rather than virus spread and plaque formation, this methodology is more accurate than the standard plaque assay to assess RSV infectivity. ***Magnification = 60X.***

We also documented transmission of the rrRSV infection to human airway epithelial cells after 48 h of incubation *in vitro* with extracts of 6 fetuses delivered from infected dams, as reflected by the appearance of red fluorescence overlapping green NGF immunoreactivity (**Figure 4**). Notably, the control cells incubated with the isotype control for the anti-NGF antibody did not show any green fluorescence, but only the red fluorescence of RSV-expressed RFP. A similar pattern was observed when the epithelial cells were incubated with extracts of newborn lungs, although the intensity of both RFP and NGF fluorescence was reduced compared to the fetal preparations.

**Figure 5:**
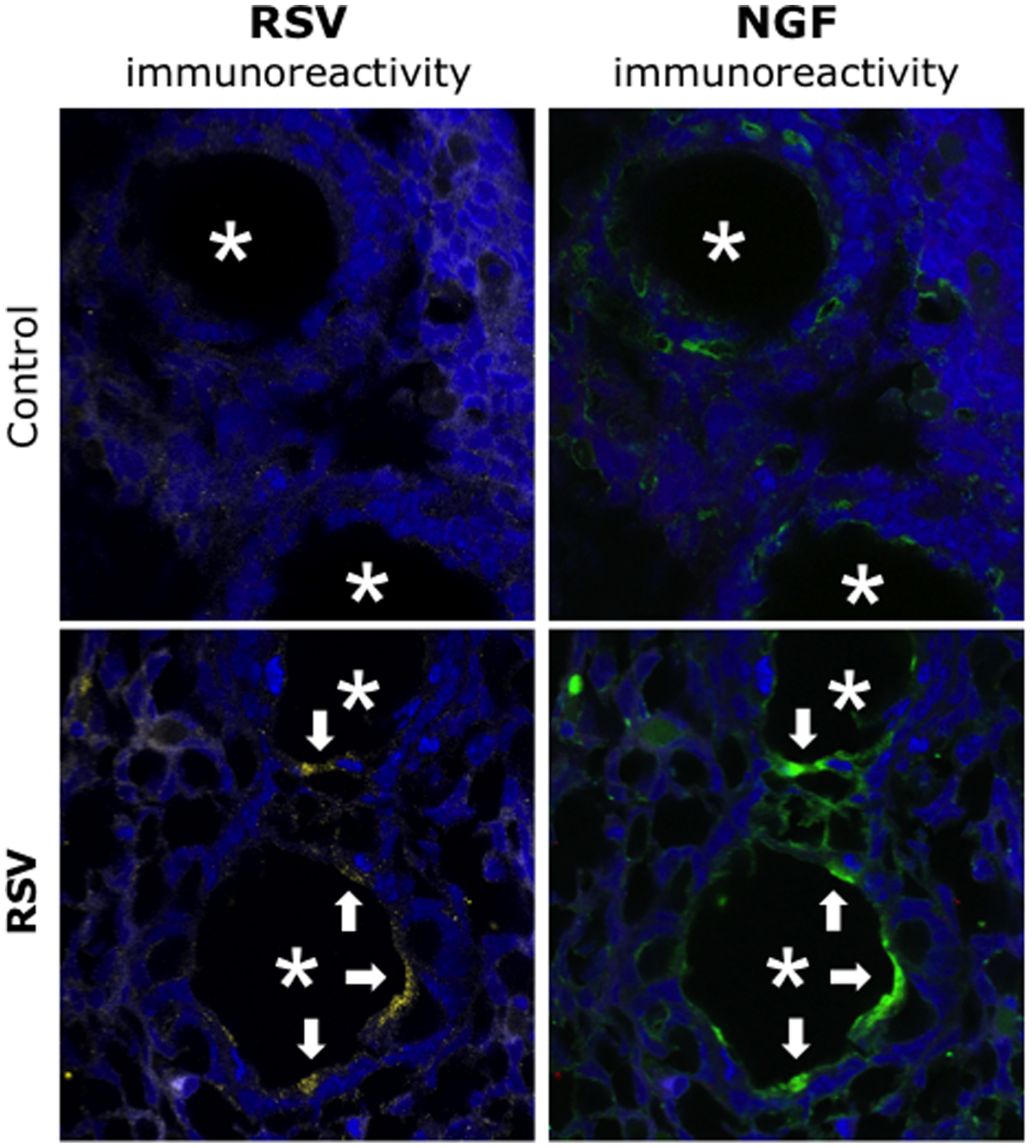
Localization of vertically transmitted RSV. Sections of fetuses delivered from dams inoculated with rrRSV or from pathogen-free controls co-stained with polyclonal antibodies against RFP and NGF. Immunohistochemical analysis of fetal airways (***asterisks***) by confocal microscopy localized increased green NGF immunoreactivity to the epithelial layer, overlapping areas of yellow RSV immunoreactivity (***arrows***). No significant fluorescence was detected in any other fetal structures. ***Magnification = 60X.***

**Figure 6:**
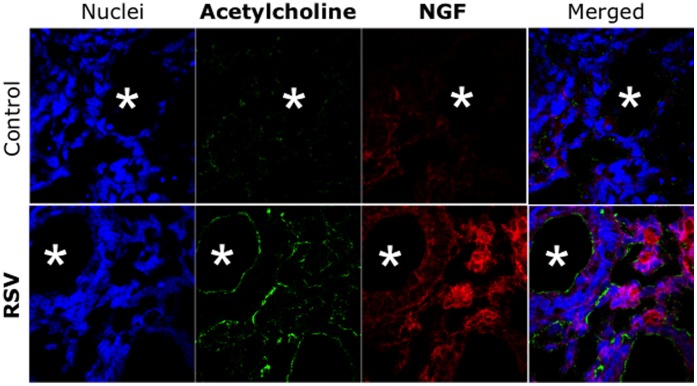
Cholinergic innervation of RSV-infected fetal airways. Sections of fetuses delivered from dams inoculated with rrRSV or from pathogen-free controls co-stained with polyclonal antibodies against acetylcholine and NGF. Immunohistochemical analysis by confocal microscopy found increased acetylcholine immunoreactivity in the subepithelial neural plexus lining the lumen of fetal airways exposed *in utero* to RSV and co-expressing NGF immunoreactivity (***asterisks***). In contrast, noradrenaline and substance P immunoreactivity was very weak in all fetuses.

Indirect immunohistochemical analysis of fetal sections by confocal microscopy localized increased NGF immunoreactivity to the epithelial layer of fetal bronchi overlapping areas of weaker but discernible RSV immunoreactivity (**Figure 5**), whereas no significant fluorescence was detected in any other fetal structures. Furthermore, we observed increased acetylcholine immunoreactivity in the subepithelial neural plexus lining the lumen of fetal bronchi exposed in utero to rrRSV and expressing NGF immunoreactivity (**Figure 6**). In contrast, noradrenaline and substance P immunoreactivity was very weak in all fetuses.

### RSV-induced Neurotrophic Dysregulation

**Figure 7:**
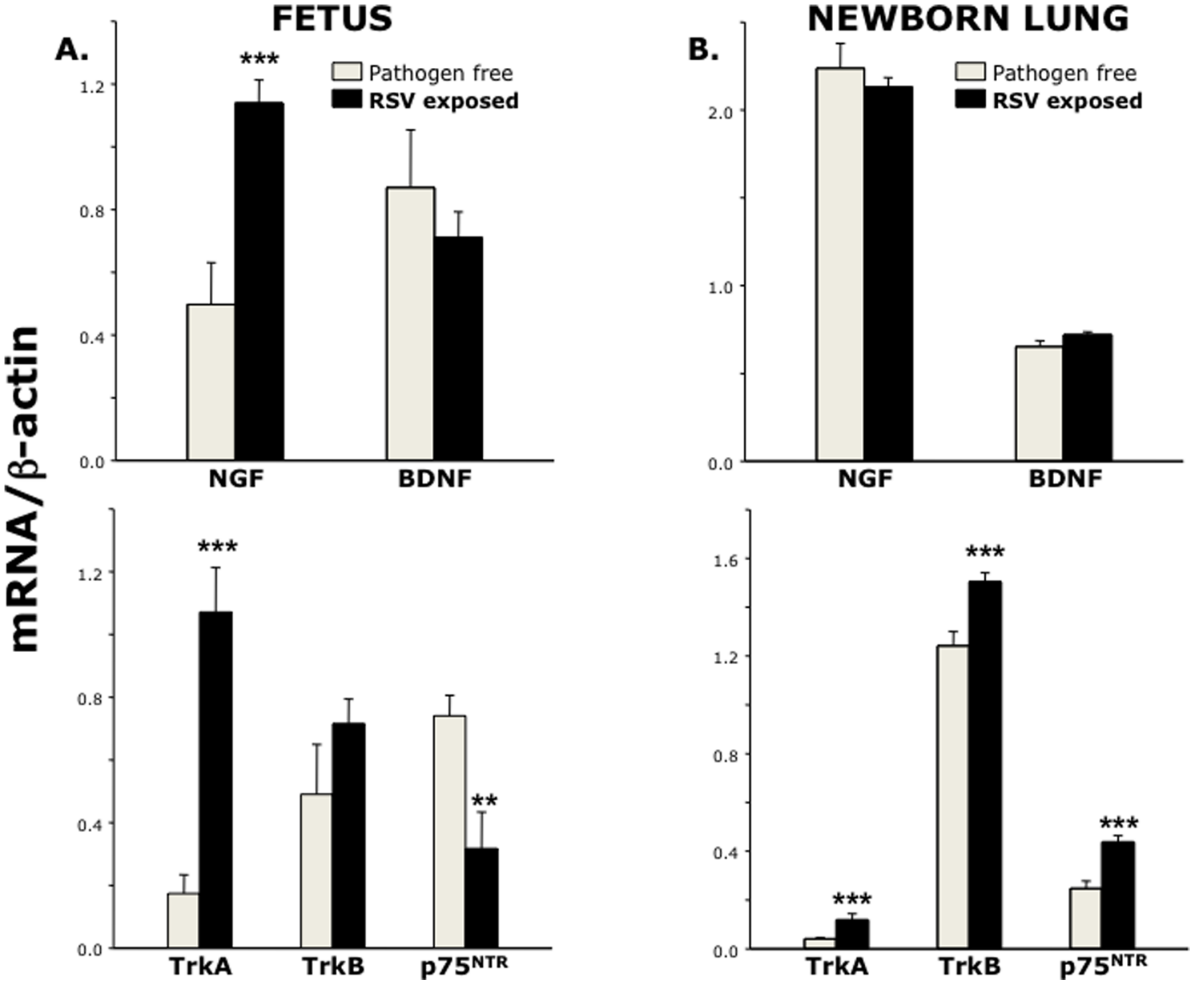
Neurotrophins expression after vertical RSV infection. *(* ***A*** *)* RT-PCR of fetuses (n = 9–10 per group) from RSV-infected dams showed upregulation of NGF and its high-affinity receptor TrkA, with downregulation of the low affinity pan-neurotrophin receptor p75^NTR^. *(* ***B*** *)* All neurotrophin receptors were upregulated in the lungs of newborns (n = 5 per group) delivered from RSV-infected dams. Data are expressed as mean ± SEM of the densitometry score normalized by the β-actin control. ** = p<0.01; *** = p<0.001 compared to age-matched controls delivered from pathogen-free dams.

RT-PCR analysis of fetal tissues exposed *in utero* to RSV (**Figure 7A**) showed a 2.2-fold higher expression of NGF gene transcripts and 6.1-fold higher expression of its cognate receptor TrkA gene compared to control fetuses harvested from pathogen-free dams (p<0.001). In the same specimens, gene expression of BDNF and its cognate receptor TrkB was unchanged (p = 0.84 and 0.21 respectively), whereas expression of the pan- neurotrophin receptor p75^NTR^ was significantly downregulated in RSV-exposed fetuses compared to pathogen-free controls (p<0.01). Postnatally, expression of NGF and BDNF genes in the lungs of newborns exposed to RSV *in utero* (**Figure 7B**) was similar to age-matched controls born from pathogen-free dams (p = 0.46 and 0.57 respectively). However, the neurotrophic receptors TrkA, TrkB, and p75^NTR^ were significantly upregulated in the lungs of newborns delivered from RSV-infected dams compared to pathogen-free controls (p<0.001). No significant increase in the expression of neurotrophic factors or receptors was detected in the lungs of weanling and adult rats born to RSV-infected dams and continuously kept in a barrier facility from birth to death.

**Figure 8:**
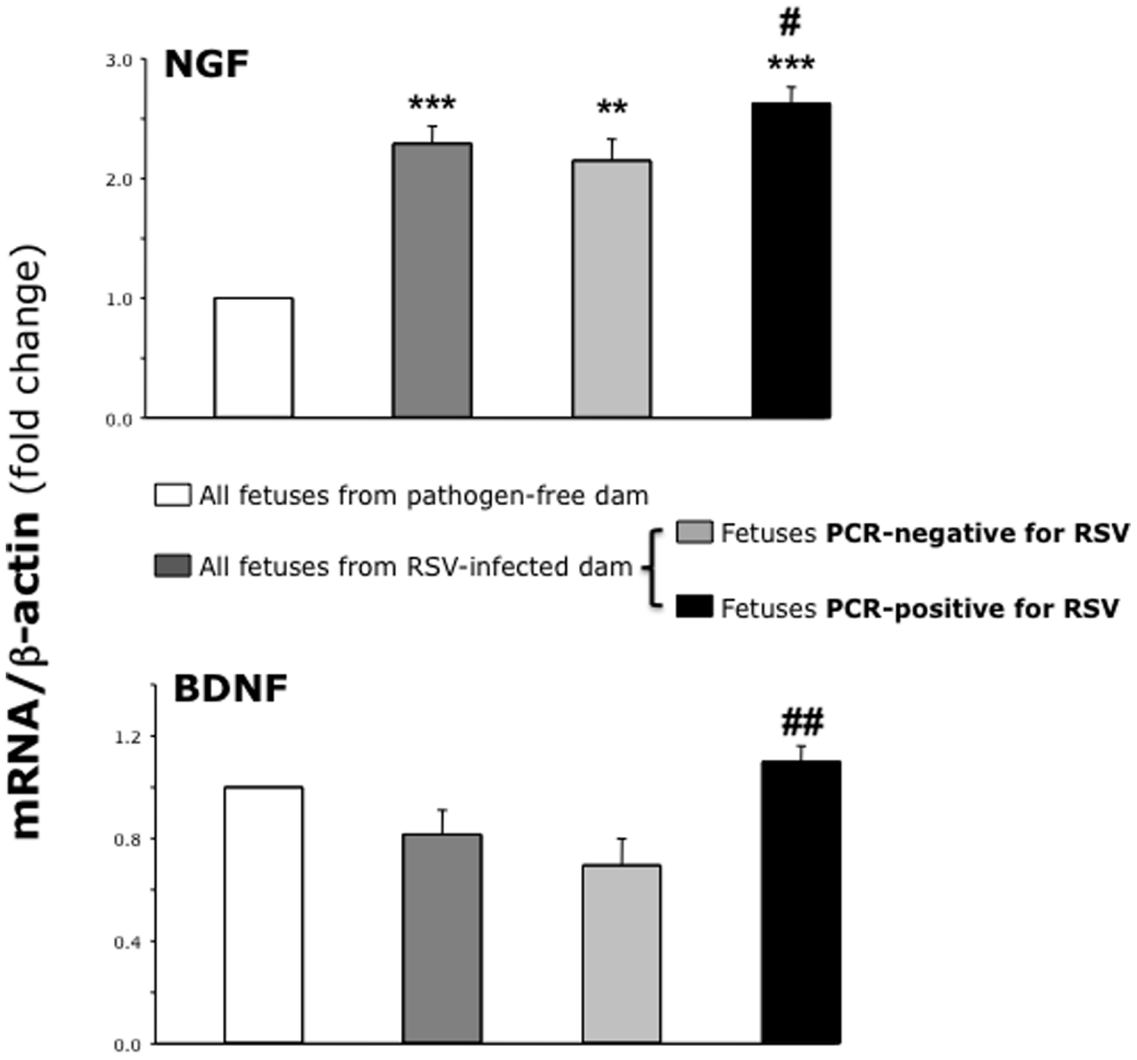
Neurotrophic factors in PCR-positive vs. PCR-negative fetuses. RT-PCR analysis of neurotrophic factors in fetuses harvested from the same RSV-infected dam. Expression of both NGF and BDNF genes was higher in fetuses RSV-positive by PCR compared to PCR-negative fetuses. However, fetuses from the RSV-infected dam had higher NGF expression compared to the control fetuses from pathogen-free dam, even if no RSV RNA could be detected. Data are expressed as the mean ± SEM (n = 9–10 per group). ** = p<0.01; *** = p<0.001 compared to fetuses delivered from pathogen-free control dam. # = p<0.05; ## = p<0.01 compared to RSV-negative fetuses delivered from same RSV-infected dam.

**Figure 9:**
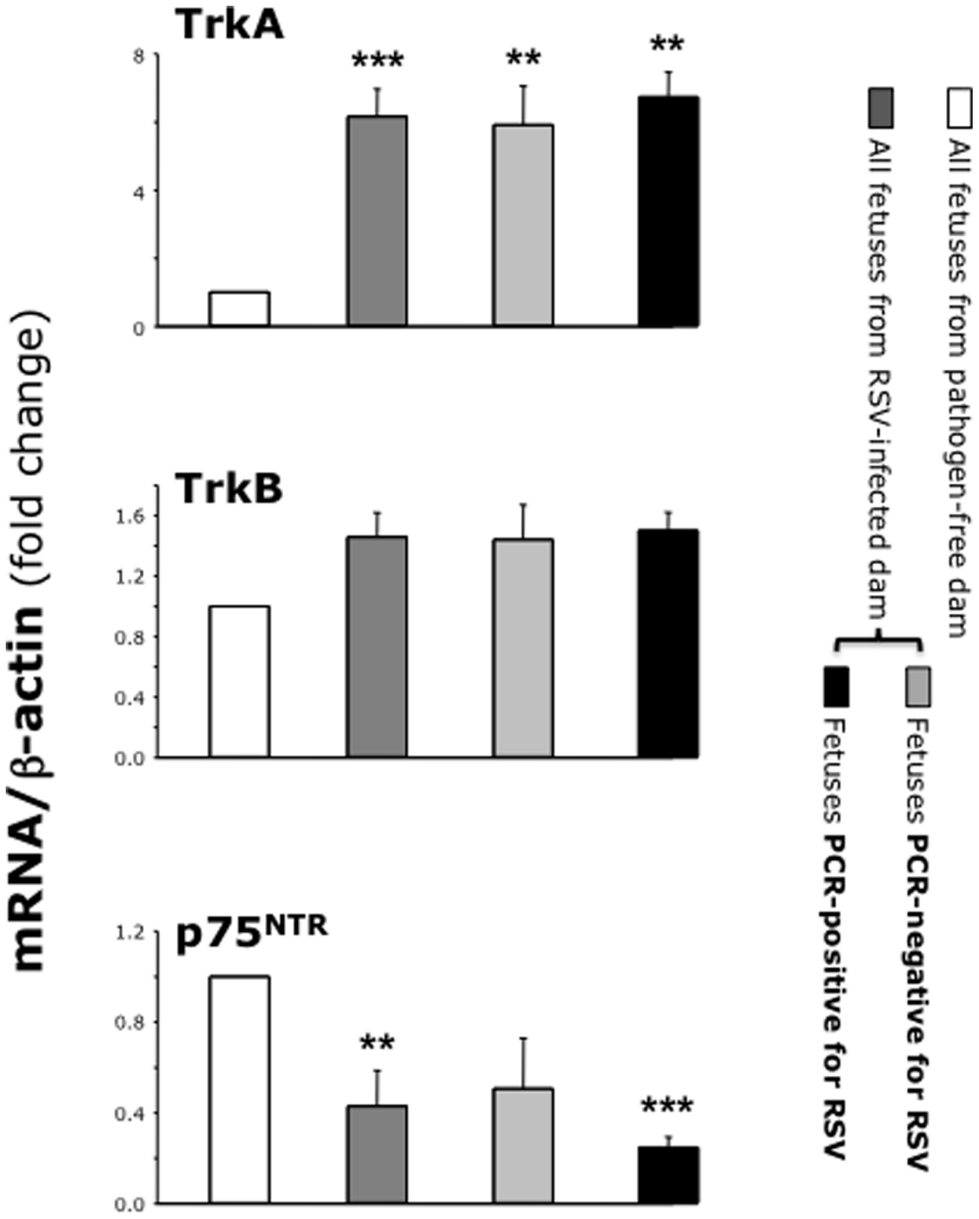
Neurotrophic receptors in PCR-positive vs. PCR-negative fetuses. RT-PCR analysis of neurotrophic receptors in fetuses harvested from the same RSV-infected dam. Fetuses from the RSV-infected dam had higher TrkA and lower p75^NTR^ expression compared to the control fetuses from pathogen-free dam, even if no RSV RNA could be detected. Data are expressed as the mean ± SEM (n = 9–10 per group). ** = p<0.01; *** = p<0.001 compared to fetuses delivered from pathogen-free control dam.

We also found important differences in the gene expression of neurotrophic factors and receptors among different fetuses harvested from the same RSV-infected dam depending on the presence or absence of PCR-detectable virus. In particular, the expression of both NGF and BDNF was significantly larger in PCR-positive fetuses compared to PCR-negative fetuses from the same RSV-infected dam (p<0.05 and <0.01 respectively; **Figure 8**). On the other hand, fetuses from RSV-infected dam had higher expression of NGF and its cognate TrkA receptor compared to fetuses from pathogen-free dam even if no virus could be detected by PCR (p<0.01; **Figure 9**).

Among other critical factors modulating lung growth and development, the only other significant change was noted for EGF, which was significantly reduced in fetuses from RSV- infected dam (p<0.05). Also for EGF, this decrease was significantly larger in fetuses PCR- positive for RSV (p<0.001) and was no longer detectable postnatally. Pre- and postnatal gene expression for all other factors tested (EGFR, TGF-β, and VEGF) showed minimal or no change after *in utero* exposure to RSV.

### RSV-induced Airway Hyperreactivity

**Figure 10:**
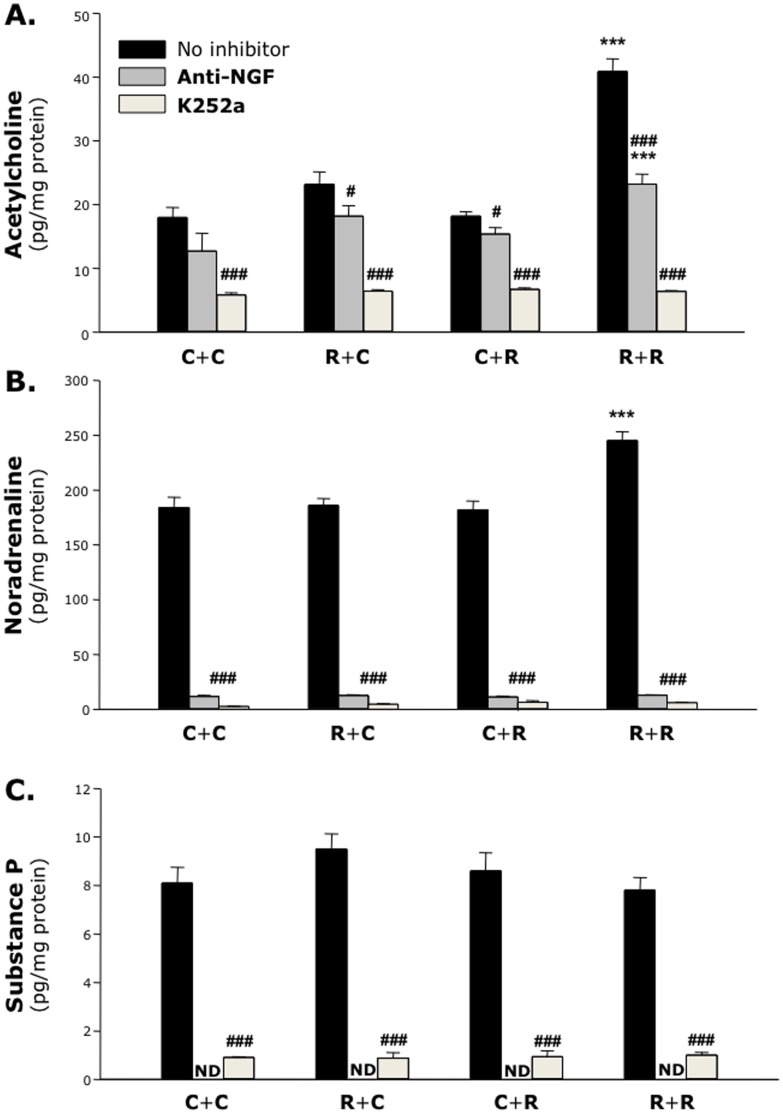
Neurotransmitters in the lung tissues. Compared to rats kept pathogen-free during and after gestation *(* ***group C+C*** *)*, cholinergic, adrenergic, and NANC innervation did not change significantly after maternal *(* ***group R+C*** *)* or neonatal *(* ***group C+R*** *)* infection. However, when pups were exposed to RSV both *in utero* and after birth *(* ***group R+R*** *)* acetylcholine expression in their lungs doubled. This effect was reduced or abolished by inhibiting neurotrophic signaling. Data are expressed as the mean ± SEM (n = 6–8 per group). *** = p<0.001 compared to C+C control. # = p<0.05; ### = p<0.001 compared to no-inhibitor control.

To explore the pathophysiologic implications of aberrant expression of neurotrophic pathways during fetal life, we measured postnatal expression of neurotransmitters in the lung tissues of pups delivered from RSV-infected dams or pathogen-free controls. Compared to rats kept pathogen-free during and after gestation (group C+C), cholinergic (acetylcholine; **Figure 10A** **, solid bars**), adrenergic (noradrenaline; **Figure 10B** **, solid bars**), and NANC (substance P; **Figure 10C** **, solid bars**) neurotransmitters did not change significantly after maternal (group R+C) or neonatal (group C+R) infection. However, when the pups from RSV-infected dams were reinfected with the same virus after birth (group R+R) acetylcholine expression in their lungs doubled compared to the control groups (p<0.001) and a smaller but significant increase was also measured for noradrenaline (p<0.001), while substance P did not change.

Confirming the neurotrophic control of acetylcholine synthesis, selective inhibition of NGF activity with a blocking antibody reduced significantly acetylcholine expression in the R+C and C+R groups (p<0.05; **Figure 10A** **, gray bars**), and this inhibitory effect was stronger in the R+R group (p<0.001); however, the effect of RSV on acetylcholine remained significant after selective NGF inhibition (p<0.001). Instead, anti-NGF virtually abolished the synthesis of noradrenaline (p<0.001; **Figure 10B** **, gray bars**) and substance P (p<0.001; **Figure 10C** **, gray bars**). Also, chemical inhibition of receptor tyrosine kinase with K252a produced maximal inhibition of all three neurotransmitters and abolished any effect of the infection (p<0.001; **Figure 10A–B** **–C, open bars**). Notably, after administration of either inhibitor the synthesis of all three neurotransmitters dropped significantly below the control values measured in the absence of neurotrophic inhibition (p<0.001).

**Figure 11:**
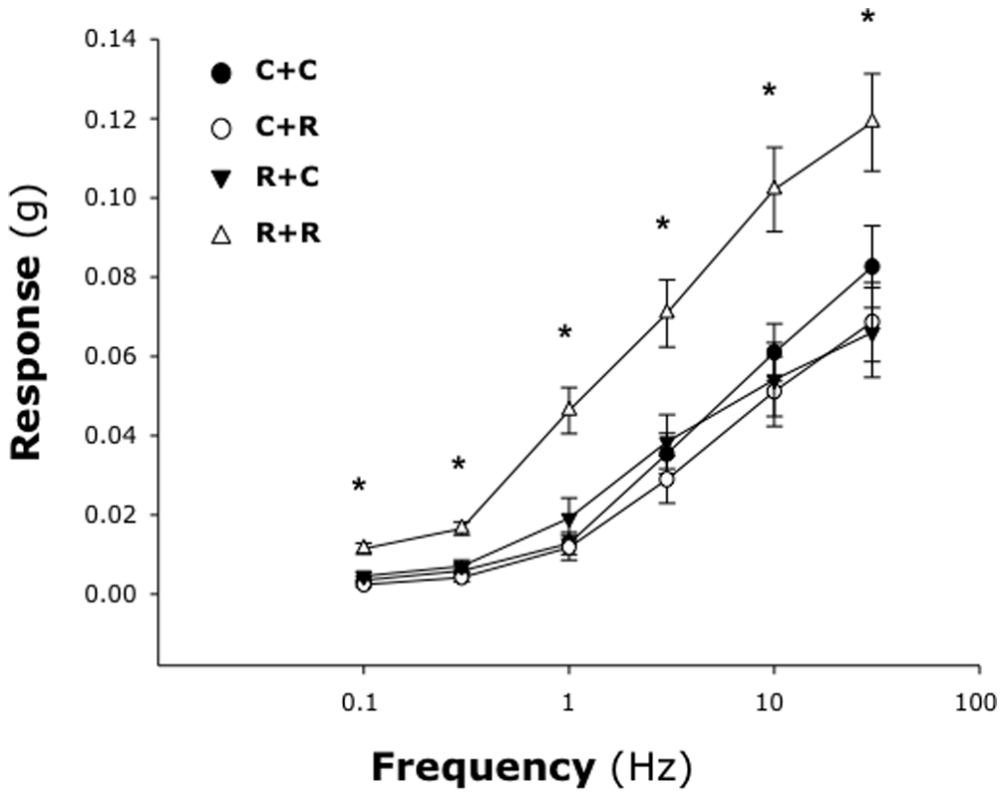
RSV-induced airway hyperreactivity in vitro. Smooth muscle contraction evoked by EFS in tracheas from pathogen-free pups delivered from pathogen-free dams *(* ***group C+C*** *)* was not affected by either prenatal *(* ***group R+C*** *)* or postnatal RSV infection *(* ***group C+R*** *)*. However, when pups delivered from RSV-infected dams were reinfected postnatally *(* ***group R+R*** *)* their airways became significantly hyperresponsive to any frequency of EFS. Data are expressed as the mean ± SEM (n = 8 per group). * = p<0.05 compared to C+C control.

Postnatal airway reactivity reflected the measured changes in cholinergic innervation both *in vitro* and *in vivo*. The smooth muscle contraction evoked by EFS in tracheas from pathogen-free pups delivered from pathogen-free dams (group C+C; **Figure 11**) was not affected significantly by neonatal RSV infection (group C+R). Also, no significant difference was found in the airways of pups delivered from RSV-infected dams (group R+C). However, when pups delivered from RSV-infected dams were reinfected postnatally (group R+R) their airways became significantly hyperresponsive to any frequency of EFS (p<0.05).

**Figure 12:**
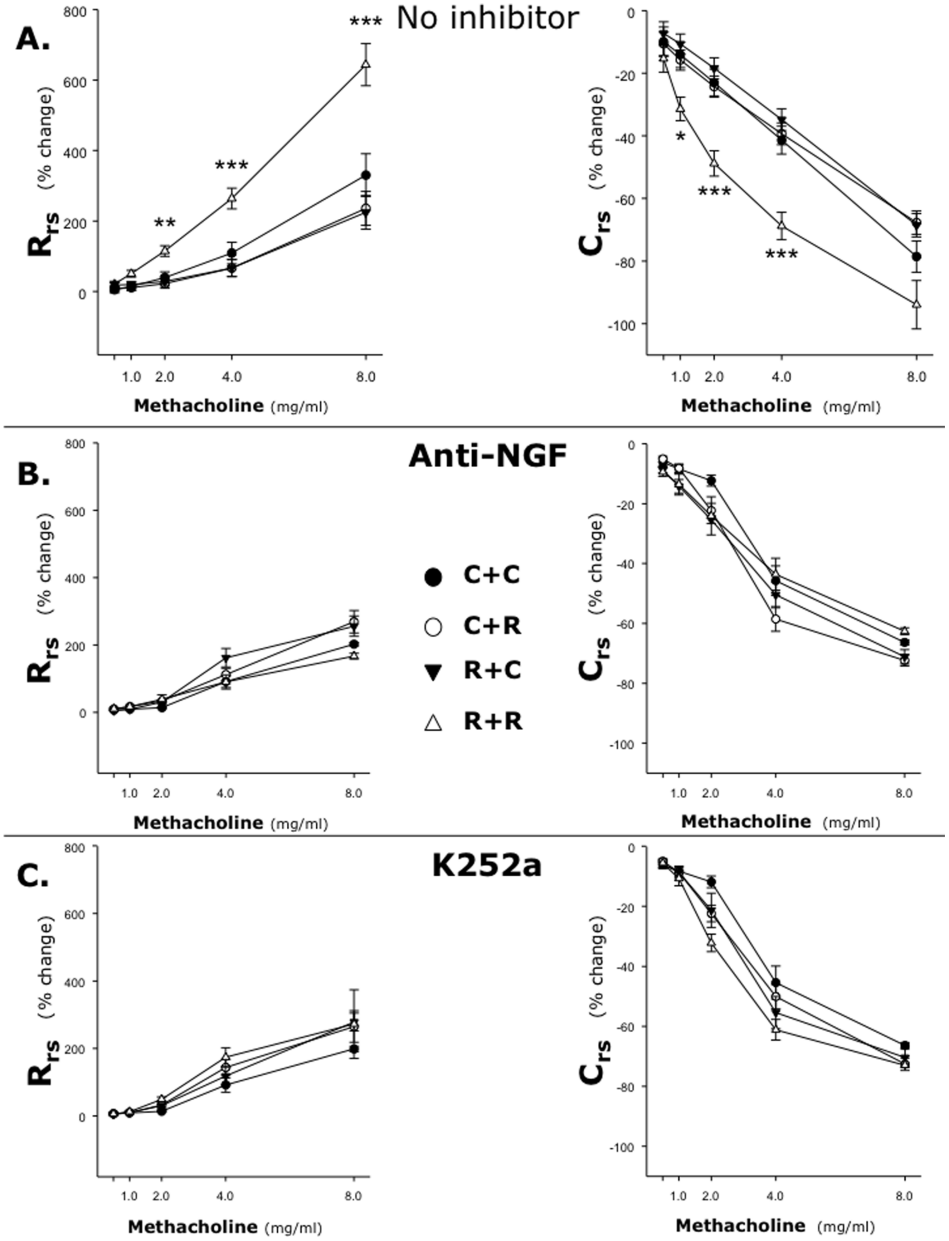
RSV-induced airway hyperreactivity in vivo. Ten-day old rats delivered from pathogen-free or RSV-infected dams were given either sterile medium *(* ***C+C and R+C groups*** *)* or RSV suspension *(* ***C+R and R+R groups*** *)*, and then challenged with inhaled methacholine 5 days later. Airway reactivity *in vivo* increased only when the exposure to RSV *in utero* was followed by postnatal reinfection with the same virus (***A***). RSV-induced airway hyperreactivity was abolished by selective immunologic inhibition of NGF activity by a blocking antibody (***B***) or chemical inhibition of receptor tyrosine kinase signaling by the specific antagonist K252a (***C***). Data are expressed as the mean ± SEM (n = 6–8 per group). * = p<0.05; ** = p<0.01; *** = p<0.001 compared to C+C control.

Similarly, inhaled methacholine concentrations >1 mg/ml increased R_rs_ and decreased C_rs_ *in vivo* only when the exposure to RSV *in utero* was followed by postnatal reinfection with the same virus (**Figure 12A**). Specifically, the increase in R_rs_ was significantly larger in the R+R group compared to all other groups at methacholine doses ranging from 2 mg/ml (p<0.01) to 8 mg/ml (p<0.001). The decrease in C_RS_ was significant in the R+R group compared to all other groups at methacholine doses ranging from 1 mg/ml (p<0.05) to 4 mg/ml (p<0.001). Statistical analysis also indicated that the individual effects of prenatal infection (p<0.001) and postnatal infection (p<0.01) were both significant within the pups of the R+R group; furthermore, there was a statistically significant interaction between the effects of pre- and postnatal infection (p<0.01).

RSV-induced airway hyperreactivity was abolished by selective immunologic inhibition of NGF activity by a blocking antibody, after which all groups responded to methacholine in a similar fashion (**Figure 12B**). A similar inhibitory effect was observed with chemical inhibition of receptor tyrosine kinase signaling by the specific antagonist K252a (**Figure 12C**). Treatment with an isotype control antibody or with the K252a vehicle had no effect on the airway response to methacholine in any of the groups (data not shown).

## Discussion

Previous studies have suggested that RSV infection is able to spread hematogenously and infect non-respiratory cells *in vivo*, as shown by the detection of viral genomic and messenger RNA in the bloodstream of infected infants [6], [29] and by the isolation of RSV in extrapulmonary sites – e.g., cerebrospinal fluid, myocardium, and liver - compatible with clinical manifestations like apnea, seizures, increased cardiac and liver enzymes that are not unusual in infants with severe RSV infection [5]. Recently, we reported the finding of RNA sequences with virtually complete homology to the RSV genome in the bone marrow of two-thirds of adult subjects and all of the pediatric subjects tested. Also, no homologous sequence was identified in the human genome database, confirming that the message was indeed of viral origin and that the bone marrow provides an extra-pulmonary target to blood-borne RSV.

Thus, albeit the clinical manifestations of RSV are predominantly circumscribed to the respiratory system, the infection has systemic implications that can affect its severity during the acute phase, as well as its long-term pathologic sequelae. Hence, we reasoned that this virus may also travel from the respiratory tract of a pregnant woman and cross the placenta to the fetus, persist in the lung tissues of the offspring, and modulate pre- and postnatal expression of growth factors, thereby predisposing to airway hyperreactivity during childhood. This became the central hypothesis of the present study, whose results show for the first time a direct transmission of RSV across the placenta into the fetuses borne by dams infected with this respiratory pathogen at midterm.

### Vertical Transmission of RSV

The virus found by PCR in the fetal tissues can replicate (as demonstrated by the virus-driven expression of RFP), infects neighboring cells (as demonstrated by the infection of airway epithelial cells exposed to fetal extracts), and exhibits the same tropism for the epithelium of the respiratory tract typical of RSV-induced infant bronchiolitis [2], [30]. The latter observation suggests that the same mechanisms of viral attachment and entry driving the tropism of this virus in early post-natal life are already functional in fetal lungs, although we cannot rule out viral replication in other tissues at levels below the threshold detected with our methodology.

The model used in this study may not take in full consideration the role of maternal antibodies [31]. As dams were not exposed to RSV before pregnancy, they had no anti-RSV antibodies when challenged with the virus. This situation may not be realistic, considering that RSV infects almost all humans by two years of age, and therefore the results presented here may overestimate the viral load that can be vertically transmitted to the fetus. However, it should be noted that RSV does not induce persistent immunity in humans, and in fact reinfection of adult healthy humans is frequent during seasonal epidemics and recognized as a common cause of community-acquired respiratory infections [1]; thus, it is conceivable that, when the mother is infected by RSV during pregnancy, the immune response may not be ready to block the virus before it enters the bloodstream and infects the fetus through the placenta.

Furthermore, when considering direct transmission of viral pathogens from mother to offspring, the pathological consequences may be significant despite preexisting maternal seroimmunity, as demonstrated by the relevant morbidity and mortality observed after transplacental infections with viruses other than RSV, such as cytomegalovirus (CMV) and pestivirus [32], [33]. Consistently, all RSV-infected dams used in our study developed measurable anti-RSV antibody titers, and yet they were often able to transfer the infection to their fetuses.

Based on previous studies, we then hypothesized that if this virus would seed in the preimmune developing fetus its antigens may be regarded as self, and this fetal immune immaturity may permit the induction of prenatal tolerance and allow the virus to persist in the lungs for variable periods after birth [34]. Consistent with this hypothesis, prenatally acquired RSV persisted into the lung tissues of part of the offspring through childhood and even adulthood, although its expression tended to decline progressively over time. Postnatal transmission by exchange of biological fluids between mother and offspring (e.g., milk or saliva) cannot explain the presence of virus in the lungs of newborns that were delivered by rigorously sterile procedures and sacrificed within 24 h. It is also unlikely to have occurred in the older rats used in our experiments because they were transferred to surrogate pathogen-free mothers immediately postpartum and were kept in a barrier facility until death.

We do recognize that placental factors and immune protection may be different in humans, and thus the efficiency and consequences for RSV infection may be different from experimental animal models; yet, exploring the biological mechanisms addressed in this study would not be ethically permissible with humans. The rat model was chosen because it has been for many years the preferred model system to study the biology of trophoblastic-uterine development, and although the organization of the rodent maternal-fetal interface is somewhat different from human primates, the structural and functional similarities are overriding. Furthermore, rodent models have been highly predictive of human pathology for several other vertically transmitted viruses, such as CMV, arenaviruses, or parvoviruses [14], and our findings in rats can explain the isolation of blood-borne RSV in the first days of life of asymptomatic human newborns [6].

### RSV-induced Neurotrophic Dysregulation

In animal models as well as in humans, postnatal RSV infections amplify neurotrophic stimulation of the peripheral afferent and efferent innervation leading to the development of aberrant airway responses [30], [35], [36]. The present study shows for the first time that fetuses vertically exposed to the infection develop similar dysregulation of neurotrophic pathways, specifically increased transcription of the genes encoding NGF and its cognate high-affinity receptor TrkA, with concomitant downregulation of the low-affinity pan-neurotrophin p75^NTR^ receptor. This is the same pattern we have shown previously in human bronchial epithelial cells infected with RSV *in vitro* [2], and in fact the strongest NGF expression in fetal lungs is localized to the epithelial lining of the bronchial tree, generally overlapping the distribution of rrRSV-expressed RFP and the cholinergic nerves of the subepithelial plexus.

Generally PCR is considered the most sensitive technique for RSV detection when compared to other standard methods like viral culture of unlabeled wild-type virus [37]. Although our data indicate that approximately one-third of exposed fetuses will harbor PCR-detectable viral genome, three clues suggest that PCR may not be sensitive enough to detect extremely low viral loads, and consequently that the actual frequency of vertical viral transmission may be even higher: 1. Fluorescent cells infected by rrRSV were detected by FACS in two-thirds of exposed fetuses; 2. PCR-negative fetuses delivered from infected dams carried low titers of infectious rrRSV that could be grown in cells and be detected by confocal microscopy; and 3. Significant changes in NGF gene expression were measured not only in fetuses that were RSV-positive by PCR, but also in PCR-negative fetuses harvested from RSV-infected dams. In the latter, however, the effect was generally smaller, suggesting a dose-dependent relationship with viral load similar to that previously shown in human bronchial epithelial cells *in vitro* [38]. We also noted a trend for TrkB increase in RSV-exposed fetuses. Although this effect did not reach statistical significance, it may still contribute to the modulation of neural development because of the low-affinity binding of NGF to TrkB and because of the increased expression of BDNF - the high-affinity ligand for TrkB - in PCR-positive fetuses.

### RSV-induced Airway Hyperreactivity

Our data indicate that pre- and postnatal RSV infections have synergistic effects on airway innervation and reactivity. After birth, acetylcholine expression in the lungs of pups exposed to RSV *in utero* was not significantly different from pathogen-free controls, and also primary neonatal infection with the virus had no effect. However, if pups already exposed *in utero* were reinfected with RSV after birth, lung acetylcholine doubled. This effect was only partially abrogated by selective NGF inhibition, but was completely abolished by tyrosine kinase inhibition with K252a. Such discrepancy may result from interactions between NGF-TrkA and BDNF-TrkB signaling, but could also reflect different tissue penetration of the low-molecular weight chemical K252a compared to the larger anti-NGF globulin.

The changes in cholinergic innervation of the respiratory tract caused by prenatal exposure to RSV result in the development of postnatal airway hyperreactivity upon reinfection with the same virus. The airway smooth muscle tone was normal in the absence of stimulation and its contraction was normal in the absence of either maternal or neonatal infection. But in pups reinfected with RSV after prenatal exposure to the virus, markedly potentiated contractile responses were measured after either electrical nerve stimulation or methacholine inhalation, suggesting the involvement of both pre- and postjunctional mechanisms. These findings are consistent and provide a plausible mechanisms to the epidemiologic evidence that early-life RSV infection – or possibly reinfection – predisposes a subpopulation of children to recurrent wheezing and asthma that typically spans through the first decade of life even in the absence of atopic phenotype [39], [40].

Possibly because of the induction of prenatal tolerance, we found limited lymphocyte activation, cytokine expression, and antibody synthesis in response to neonatal infection after RSV exposure *in utero* (data not shown). However, it should be noted that these pups were sacrificed only 5 days after reinfection, i.e., before the full activation of adaptive immune responses that require approximately 21–28 days and are the focus of ongoing studies looking at the chronic phase of postviral airway dysfunction. Also, based on our recent finding that epithelial NGF expression is controlled via epigenetic mechanisms [38], we are testing the hypothesis that prenatal RSV infection interferes with the NGF promoter via suppression of specific miRNAs (especially miR-221) and selective demethylation, and that these effects are maintained by the postnatal persistence of virus in the developing lungs.

### Conclusions

In our experimental model, RSV was able to spread across the placenta from the respiratory tract of the mother to the fetus, and was also detected postnatally in the lungs throughout development and into adulthood. Vertical RSV infection was associated with dysregulation of critical neurotrophic pathways during fetal development, leading to aberrant innervation and increased airway reactivity after postnatal reinfection with RSV. This study challenges the current paradigm that RSV infection is acquired only after birth and shifts the attention to the prenatal effects of the virus, which may result in more severe and lasting consequences by interfering with critical developmental processes.
